# Behavior of Weld to S960MC High Strength Steel from Joining Process at Micro-Jet Cooling with Critical Parameters under Static and Fatigue Loading

**DOI:** 10.3390/ma14112707

**Published:** 2021-05-21

**Authors:** Tadeusz Szymczak, Bożena Szczucka-Lasota, Tomasz Węgrzyn, Bogusław Łazarz, Adam Jurek

**Affiliations:** 1 Department of Vehicle Type-Approval & Testing, Motor Transport Institute, Jagiellońska 80, 03-301 Warszawa, Poland; tadeusz.szymczak@its.waw.pl; 2 Faculty of Transport and Aviation Engineering, Silesian University of Technology, 40-119 Katowice, Poland; bozena.szczucka-lasota@polsl.pl (B.S.-L.); boguslaw.lazarz@polsl.pl (B.Ł.); 3 Novar Sp. z o. o., Towarowa 2, 44-100 Gliwice, Poland; adam.jurek@novar.pl

**Keywords:** high-strength steel, components, joining, transport, micro-jet, welding, mechanical resistance, parameters, microstructure, mini-specimen, fracture, fatigue limit

## Abstract

The paper is focused on testing the weld of the S960MC steel produced at the micro-jet cooling under static and fatigue loading at critical parameters. This kind of material was in the form of a sheet with a thickness equal to 2 mm. The joint was obtained using three different types of welding wires: EDFK 1000, Union NiMoCr and Union X96 at the same parameters of the process. The joints were examined using non-destructive and destructive tests. The results from non-destructive experiments enable us to assess the quality of the welds directly before the joining process. In contrast, the destructive one allows following welds behavior under different loading conditions with their critical parameters. The bending experiments confirmed the good plastic properties of the weld, expressed by no cracks in the region tested in many variants of the joint manufactured. The results from static tests indicated a significant reduction of mechanical parameters of the weld in comparison to the base metal, expressed by 50% differences. Fatigue data have enabled us to follow the welding behavior at the increasing amplitude of axial stress up to fracture at constant amplitude value covering the following values of stress 650 MPa–100 MPa. Variations of total energy are presented at different values of several cycles up to fracture. Fracture regions are collected for analysis of the joint region features under cyclic loading. They have indicated differences in weld cracking depended on the stress level. Finally, the Wöhler S-N curve of the weld was determined, indicating the value of the fatigue limit of the weld tested, i.e., 100 MPa. The weld at the Union NiMoCr welding wire was indicated as the joint having the highest resistance on static and fatigue loadings.

## 1. Introduction

High-Strength Steels (HSS) belong to modern metals used in a lot of branches of the industry concerning their mechanical properties, such as ultimate tensile strength and yield stress, which are higher compared to typical structural materials [1,2]. Among them, the following material types can be indicated: Strenx [1,3], Docol [4,5], Amstrong Ultra [6] and Optim QC [7]. Their application is very wide, and it is selected based on comparing the value of stress due to operational conditions and mechanical parameters at microstructure features [1,2]. Therefore, they are employed for manufacturing different components called: car bodies, stringers [8], frames [9], bumper reinforcement and door beams [5], Rear Underrun Protective Device [1,10], booms [6,7], platform [8], tippers and aerial platforms [6].

Until now, unalloyed steel with a minimum yield strength of 355 MPa and minimum impact strength KV-20 = 27 J was used for the construction of the container part of the container-type vehicle [11]. Due to low strength, the walls of the containers were massive, with a thickness of up to 5 mm. Recently, attempts have been made to use high-strength steel (e.g., S960 grades) for the construction of containers, which has a strength of 2.5 times greater, which makes it possible to significantly reduce the thickness of the container wall, and thus the entire weight of the container [12]. This is very important in transport, as reducing the weight of the container will allow you to carry more loads. It is a very economical solution [13]. Worth noticing, the production process for elements made of HSS materials is very different, and it can be conducted using welding, screwing, or bolting techniques. Moreover, in the case of the last connection methods, the sub-components used for the joining have certificates, while the quality of the first one depends on the qualifications of the welding group. This problem is considered by a lot of research teams [9,14], which examined parameters of the joining technique or modified ones applying additional sub-elements such as micro-jet cooling device [15,16] or hybrid welding process [17,18]. These efforts are transferred for various kinds of high-strength steel focusing on the S960MC [15,17,18], which is an attractive kind of metal because it is produced in a lot of forms such as sheets, tubes and plates [1,6]. The Strenx 960MC is very good weldable, cold-formed and machined by cutting. Therefore, typical applications are represented by advanced lifting devices such as mobile cranes and lighter transport solutions and components [3,6]. They indicate the steel and their joints can be subjected to various types of loading, including: static and cyclic, which influence mechanical parameters important for engineering practice and inspection stages for a correct designing and service life, respectively. Therefore, different tests are used for determining the steel reaction [19]. They follow the behavior of the welded zones with different filler materials up to fatigue limits indicating its value close to 100 MPa at a very narrow stress range represented by the following values 100–300 MPa, [18]. Moreover, taking these data, differences between the role of metal used for filling can be easily indicated. Besides fatigue tests, hardness experiments are still used as the fundamental probe for assessing the quality of a welded joint made of the S960MC steel [18]. This method is very helpful at the calibration of the welding process as well as the hybrid joining technique. Moreover, the technique is very typical; nevertheless, some differences between welded regions can be easily indicated [18]. This type of results enables to follow concludes on the quality of joint if sufficient numbers of measurement regions are collected [20]. This approach can also be evidence in [17,21], which presents data from micro-hardness tests [17] on the joint manufactured at the Gas Metal Arc Weld (GMAW). The same sentence, as it has been formulated in the case of the hardness probe, can be addressed to impact test, which has enabled indicating advantages of the laser and laser welding process, expressed by the following values of accumulated energy reaching 57 J and 49 J, respectively. The same kind of test was also used for differencing features of the S960MC welds produced by various types of the process [22]. In this case, the experiment type was very effective and has enabled us to distinguish variations of impact toughness. Some authors [23] have used a combined approach such as fracture toughness results to predict fatigue endurance of a weld of the S960MC. They have presented a fatigue curve at stress value close to data shown in [18], indicating good agreement between the method and experimental efforts.

Welding S690 steel grades is not easy. For making joints with a thickness of about 6 mm, it is absolutely necessary to use preheating and control the inter-stitch temperature. Joints made of these steels have low fatigue strength and low relative elongation. The softening of the HAZ (Heat Affected Zone) is one of the major issues of HSS welding [24,25]. There is no information in the technical literature on the fatigue strength of welded joints made of S960 steel grade. Thin-walled joints are a major welding problem, as they can be prone to welding cracks.

Pipe joints made of HSS steel with variable wall thickness were tested. It has been shown that a thick-walled joint has better fatigue properties than a thin-walled joint due to the reduction of the stress concentration factor. In fact, the presence of fatigue cracks or otherwise induced defects in such connections poses a potential safety risk to the structure. The authors found the possibility of cold cracks, especially in joints of greater thickness, where they recommend using preheating. It is also related to the higher hydrogen content in the joint obtained without preheating [26,27].

In welding AHSS (Advanced High Strength Steel) steels, there is a need to modify the technology to improve the strength and plastic properties of the joint. This is due to the slightly different metallographic structure of the weld and the base material (expanded ferrite and martensite in the weld). For this purpose, for the first time, it was decided to test the micro-jet cooling method during S960MC welding (research gap), which can lead to the fragmentation of the joint structure, which is related to the improvement of the mechanical properties of the joint. For the first time, fatigue tests of such a joint were performed, which are the most important information leading to the verification and validation of the new design and technology.

The aim of the article was to develop a material and technological solution enabling the production of a thin-walled structure made of difficult-to-weld steel, characterized by the best mechanical properties. Therefore, in the article, it was decided to very carefully check the possibility of MAG (Metal Active Gas) welding S960MC steel using various parameters, materials and welding methods. It was also decided to check the possibility of welding S960MC steel to obtain the best mechanical properties. It was decided to test three electrode wires with a variable content of carbon and other alloying elements, and it was decided to test the use of two different shielding mixtures in the MAG welding process. Independently, it was decided to check the possibility of making a thin-walled structure of the S960MC steel joint with the use of micro-jet cooling, believing that this type of cooling would allow for the fragmentation of the dominant martensitic and ferritic structures. It was decided to perform numerous non-destructive and destructive tests to determine the most favorable welding and cooling parameters of the micro-jet, which allows the best mechanical properties of the joint to be obtained. Taking into account the results discussed above, the two research paths for examining the S960MC weld are evidenced. The first one is represented by typical experiments such as hardness and fatigue tests but at a narrow range of experimental procedures, while the second manner is expressed by complex approach, i.e., theoretic-experimental. From the practical and scientific point of view, these manners are suited for selected loading regimes, without details on the energetic aspects of the fatigue process as well as cases at high value of stress, including an increase of amplitude related to the fractured moment. The most important point of the research was to check the fatigue strength after welding S960MC steel. For this purpose, the size of the samples was specially designed, and fatigue strength tests were performed on very sensitive equipment.

In addition to the use of micro-jet cooling, the type of shielding gas plays an important role. Only the use of the Ar–CO_2_ mixture allows obtaining the correct joints. Further research focused on the evaluation of joints made with three different welding wires. M21 (Ar-18% CO_2_) cover and micro-jet cooling were always used. Tensile and fatigue tests were performed. The influence of the selection of the electrode wire on the mechanical properties of the joint was analyzed. The wires had a comparable chemical composition but differed slightly in carbon content. It was believed that the carbon content of the wire could affect the mechanical properties of the joint: both tensile strength and fatigue strength.

## 2. Materials and Methods

### 2.1. Specimen for Welding and Parameters of the Joining Process

**Figure 1:**
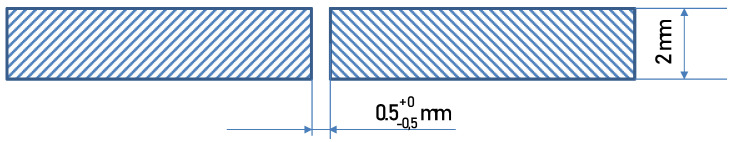
Preparation of the element for metal active gas (MAG) welding with micro-jet cooling.

Welded butt joints of S960MC steel with a thickness of 2 mm, length 200 mm and width 300 mm were made. The MAG (Metal Active Gas) welding process was used according to the standard requirements (EN 15614-1). The preparation of the material for single-stitch welding is shown in Figure 1.

It was decided to make test joints using two shielding gases: CO_2_ and gas mixture M21, i.e., Ar + 18% CO_2_ (according to the PN-EN 14175 standard). All of the sheet metal samples were welded with three different electrode wires (ED-FK 1000, Union NiMoCr and UNION X96) with a similar chemical composition but with a slightly increasing carbon content in each of them (0.08% C; 0.09% C; 0.11% C):

- EN ISO 12534-A G 69 5 M Mn4Ni1.5Cr-Union (Voestalpine Böhler Welding, Hamm, Germany) NiMoCr (C 0.08, Si 0.6, Mn 1.7, Cr 0.2, Mo 0.5 and Ni 1.5);
- EN ISO 16834-A G 89 6 M21 Mn4Ni2CrMo-ED-FK 1000 (FLIESS, Duisburg, Germany) (C 0.09, Si 0.8, Mn 1.8, Cr 0.31, Mo 0.55 and Ni 2.2);
- EN ISO 16834-A G 89 5 M21 Mn4Ni2.5CrMo-UNION X96 (Voestalpine Böhler Welding, Hamm, Germany) (C 0.11, Si 0.78, Mn 1.9, P 0.01, S 0.009, Cr 0.35, Mo 0.57, Ni 2.23, V 0.004, Cu 0.02, Ti 0.057, Zr 0.001 and Al 0.002).

The chemical composition of the three selected electrode wires was comparable. A slight difference in the carbon amount was noted. The carbon content influences the steel weldability and tensile strength. There were also slight differences in the chemical composition between the base material and the electrode wires. Molybdenum and nickel, which are not present in steel, were additionally introduced in the electrode wires. This is conducted in order to increase the plastic properties of the joint. Vanadium and copper in both the base material and the filler wires are low. However, there are noticeable differences in the content of aluminum, especially titanium, which is responsible for the precipitation strengthening of the joint [28].

In the initial stage, the MAG welding parameters were established: current 95–105 A, arc voltage (17–22 V) and welding speed (250–350 mm/min). Observations were made 48 h after welding to ensure that no cold cracks would form (by the recommendations of the standard PN-EN 1090-2). Welding tests were performed with the assumed gas flow of 14 L/min. The input energy during the welding of thicker sheets was below the recommended value of 4 kJ/mm. All welding tests were carried out without preheating. The best results (correct joint form) were put in Table 1.

The chemical composition and mechanical properties of the S960MC steel are presented in [3]. The gap between sheets was varied in the range 0–1 mm. The correct results were obtained for the gap equal to 0.5 mm, Figure 1. This case was only taken for further investigation. The most important other parameters of the welding process are given in Table 1.

S960MC steel was welded using micro-jet cooling. Micro-jet cooling parameters were varied in the following way:

- micro-jet gas: argon;
- stream diameter: 60 and 70 μm;
- gas pressure: 0.6 and 0.7 MPa.

Non-destructive and destructive tests were used to assess the quality of the welds according to the research plan presented in Table 2.

### 2.2. Details of Static and Fatigue Tests

**Figure 2:**
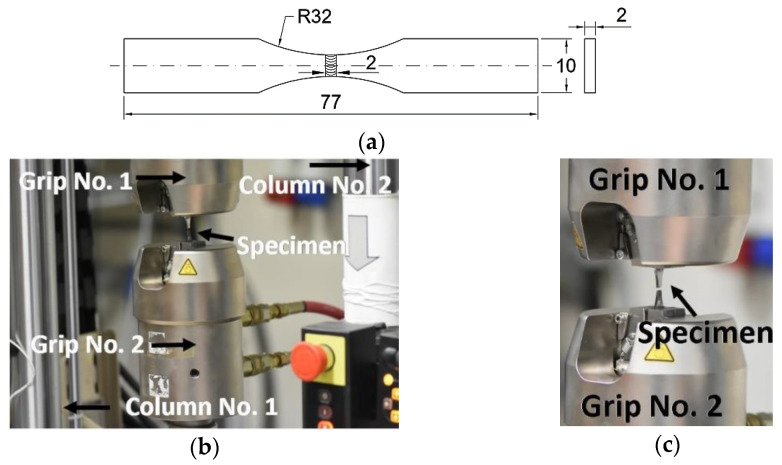
U-notched mini-specimen in the 8874 Instron testing machine: (**a**) shape and dimensions (in mm) of the specimen, (**b**) general view and (**c**) mini-specimen and grips.

Concerning directly examining the weld at the width of 0.5 mm under loading, the used specimens were in a mini-scale. They possessed the U-shaped working zone with the welded region in the middle of the zone considered, Figure 2a. This kind of specimen has enabled examining the weld directly under loading and following easy degradation of the region tested. Moreover, the specimen shape reduces the influence of the surface quality of the surrounding regions on results. All tests were carried out at room temperature using the 8874 Instron servo-hydraulic testing machine, Figure 2b,c. Mini-specimens were directly mounted in the grips and loaded. For the small distance between the upper and lower grip, the extensometer technique was not used. The following sensors of the testing machine were employed: load cell and displacement straight edge.

**Figure 3:**
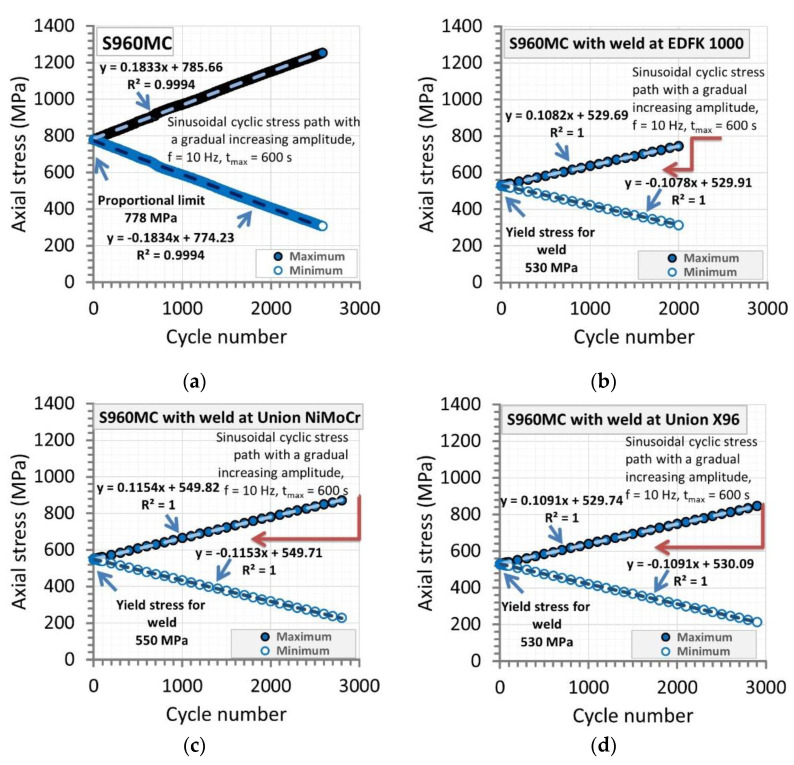
Courses of minimum and maximum values of axial stress amplitude from a test on the S960MC (**a**) and micro-jet cooling welds of the material manufactured at the following welding wires: (**b**) EDFK 1000, (**c**) Union NiMoCr and (**d**) Union X96.

For the micro-jet cooled welds, the fatigue experiments were used to determine some differences between the joints. The results from these tests were selected for extending the knowledge on the behavior of joints type examined, collecting differences for indicating the strongest and weakest weld. Therefore, the fatigue test contained two stages. The first one, in the case of the base materials, was designed for values of proportional limit and ultimate tensile strength (Figure 3a). In the case of the welds, the yield stress and ultimate tensile strength were taken as an initial and final point for the cyclic sinusoidal stress signal (Figure 3b–d). The value of ultimate tensile strength was indicated as the amplitude at the last point of the cyclic course defined by a value of time (Table 3).

The selected value of time to fracture under the stress cycles was represented by 600 s. This was directly used in the Envelop section for cyclic signals in the Wave Matrix software of the 8874 Instron servo-hydraulic testing machine. In all cases, the frequency was equal to 10 Hz. In all experimental efforts, the value of yield stress was reached at the same time; next, the stress cycles were conducted at an increasing value of stress amplitude at the minimum and maximum values in a range of time 0–600 s (Figure 3). The tests were conducted up to specimen fracture starting from the value of yield stress.

**Figure 4:**
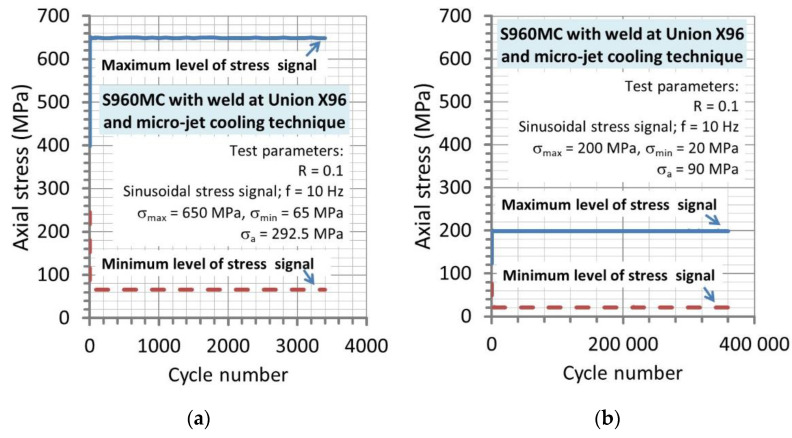
Maximum and minimum values of stress signals used for determining the Wöhler curve of the joint from the welding process supported by the micro-jet technique at the Union X96 welding wire: (**a**) the highest and (**b**) the lowest stress range applied in the test.

Fatigue tests on the Wöhler’s curve were conducted at stress signal in the form of a sinusoidal function at frequency 10 Hz; stress ratio R = 0.1 and values of amplitude as follows: 650 MPa, 600 MPa, 550 MPa, 500 MPa, 400 MPa, 300 MPa, 200 MPa and 100 MPa (Figure 4).

## 3. Results and Discussion

### 3.1. The Results of Non-Destructive Tests

Non-destructive test (NDT) results are presented in Table 4. The results of NDT tests allow for a preliminary assessment of the quality of the weld. They expressed differences in the weld quality and indicate the NDT is very important for determining the quality of the zone examined because defects such as cracks can be evidenced.

**Figure 5:**
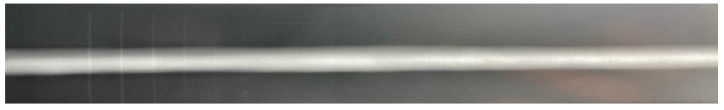
Correctly made joint after welding with micro-jet cooling.

**Figure 6:**
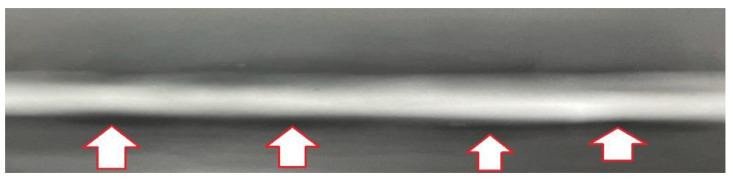
Welding defects (cracks) and incompatibilities after classic MAG welding without micro-jet cooling.

Based on the results of non-destructive testing, the samples in Table 4 were marked as “✓”—positive and “**×**”—negative. Examples of non-destructive testing results are shown in Figure 5 and Figure 6. Figure 5 shows a positive result recorded for sample UXm3. The quality of the sample was on level B. No cracks and welding incompatibilities were found in the analyzed joint. Figure 6 shows a negative result. The result was obtained for sample UXm1, which is characterized by welding incompatibilities.

MAG welding in CO_2_ shield is more oxidizing than welding in an Ar-18% CO_2_ shield. Welding in a CO_2_ shield corresponds to the oxygen content in the weld at the level of 500 ppm, which translates into the larger size of non-metallic inclusions that may initiate cracks. Welding in the Ar–CO_2_ mixture shield is less oxidizing; it leads to the formation of smaller non-metallic inclusions that may, to a lesser extent, cause cracks in the joint. The classification of welding processes in terms of the oxygen content in the weld and its justification was presented in 1999 [29,30].

Based on the analysis of the table data, it can be noticed that the M21 mixture (Ar + 18% CO_2_) is much better than CO_2_. From the analysis of the results presented in Table 4, it follows that the test was carried out correctly; the evaluation of the tests is positive only in some cases when micro-jet cooling was not too intense and not too weak. For further tests (destructive test—DT), only those joints that met these requirements were taken into account:

- made with the use of micro-jet cooling;
- no cracks were observed.

Therefore, samples with the following determinations were taken into account (UNm3, UNm4, EDm3, Edm4, UXm3, UXm4 and UNc4, EDc3).

### 3.2. Data from the Bending and Hardness Tests

The test results of bending tests are summarized in Table 5. The process was correct only with medium-power micro-jet cooling because no cracks and other disconformities were found in the samples tested. It reflected the high-quality level of the joint, and it was confirmed in comparison to the regimes of the PN-EN ISO 5817 standard [31] on a crack length ≥ 0.5 mm at the highest requirement. Sample section dimension: 2 mm × 20 mm.

The bending tests confirmed that after welding with micro-jet cooling, it is possible to achieve joints with good plastic properties, as measured by the absence of cracks. In addition to the use of micro-jet cooling, the type of shielding gas plays an important role. Only the use of the Ar–CO_2_ mixture allows obtaining the correct joints.

### 3.3. Metallographic Examination and Hardness

**Figure 7:**
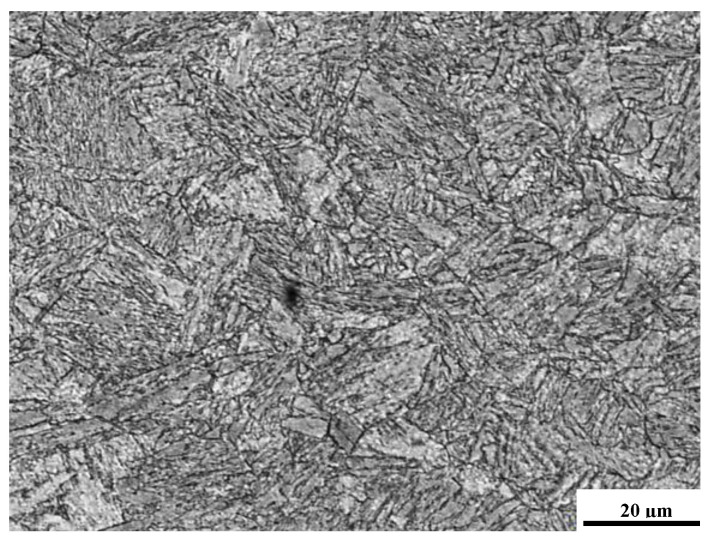
The microstructure of the joint (UXm3) with visible martensite and course ferrite (LM—Light Microscopy).

Microstructure observations were carried out on the Neophot 32 light microscope (Carl Zeiss Jena, Jena, Germany). The structure is dominated by martensite and ferrite. Figure 7 shows the structure observed in the UXm3 sample. Generally, martensite and coarse-grained ferrite were observed.

**Figure 8:**
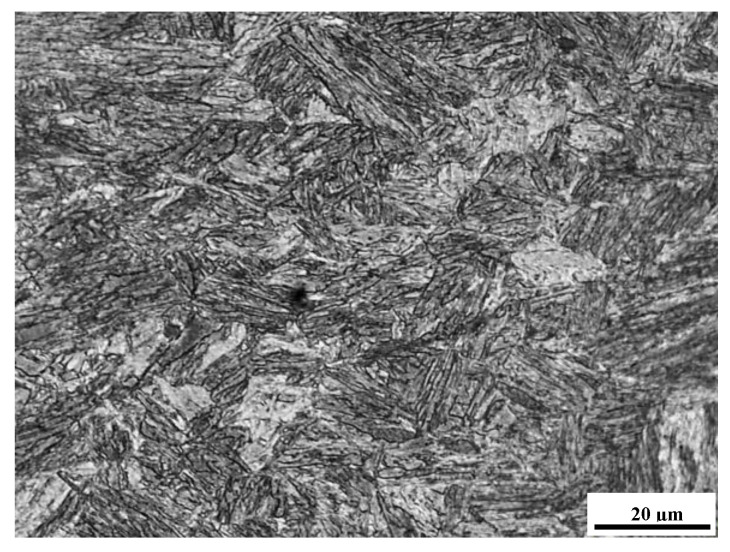
The microstructure of the joint (UXm3) after welding with micro-jet cooling with visible martensite and fine-grained ferrite (LM).

Only the application of micro-jet cooling after MAG welding made it possible to obtain more fragmented phases: martensite and ferrite (shown in Figure 8). This is due to micro-jet cooling during welding, which limits phase growth during the austenitic transformation. Other authors also draw attention to the possibility of grain fragmentation because of the use of micro-jet cooling [28,32].

After microscopic observation, it was decided to perform hardness tests. It was made for the same samples. The results are presented in Table 6.

The table data shows that a more favorable and even distribution of hardness in all tested zones is for a joint made with micro-jet cooling.

### 3.4. The Steel and Its Micro-Jet Cooled Joint at Different Welding Wire in a Static Test

**Figure 9:**
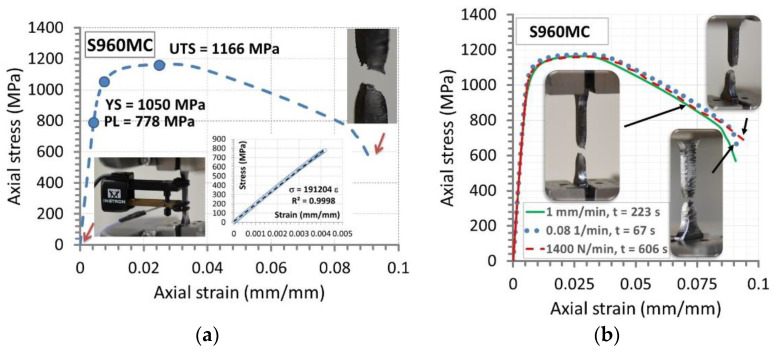
The tensile characteristic of S960MC steel (**a**) and its variations (**b**) due to three different signals used in the tensile test: displacement at velocity of 1 mm/min, strain at 0.08 1/min and stress reaching 117 MPa/min, proportional limit (PL) = 778 MPa, yield stress (YS) = 1050 MPa, ultimate tensile strength (UTS) = 1166 MPa and elongation 8.75%.

S960MC steel appears as the material having elastic-plastic with hardening and unstable regions (Figure 9). The proportion between ultimate tensile strength (UTS) and yield stress (YS) reached the value of 1.11, expressing the small distance between the mechanical parameters considered. From the practical point of view, this feature indicates an inspection of the element made of this kind of steel if the stress has equaled yield stress during operation. Another important sign in the S960MC behavior under tensile stress is evidenced in a region of the unstable section, which dominates in 70% comparing to the elastic and elastic-plastic with hardening zones. This informs the steel can carry loading at a long moment after a value of stress exceeds ultimate tensile strength. Taking the long unstable section, the steel can be easy to diagnose at high values of stress without any difficulties and risks for researchers, engineers and diagnostics teams. This sentence is also confirmed by a value of stress at the steel rupture, i.e., 600 MPa, indicating two times difference comparing to the value of ultimate tensile strength and distancing the fracture up to a value of a relative elongation of 9.5%. The behavior of the steel follows advantages concerning the application of this kind of material in a tri-axial stress state because the necking effect is significant, and this prevents brittle cracking.

The advantages of the S960MC steel are captured at tensile stress conducted at various types of control signals represented by the following velocity values of displacement, strain and stress signals such as 1 mm/min, 0.08 L/min and 117 MPa/min, respectively (Figure 9). As it can be noticed, the stress–strain curves follow the same course, which expresses insensitivity of the steel tested on the type of tensile signal and the time up to fracture related to the control kind used. This enables to sentence the mechanical parameters of the steel are stable for the wide range of loading signals and their parameters, extending the application of the material examined to operational conditions with different velocity diverse by even 10 times.

**Figure 10:**
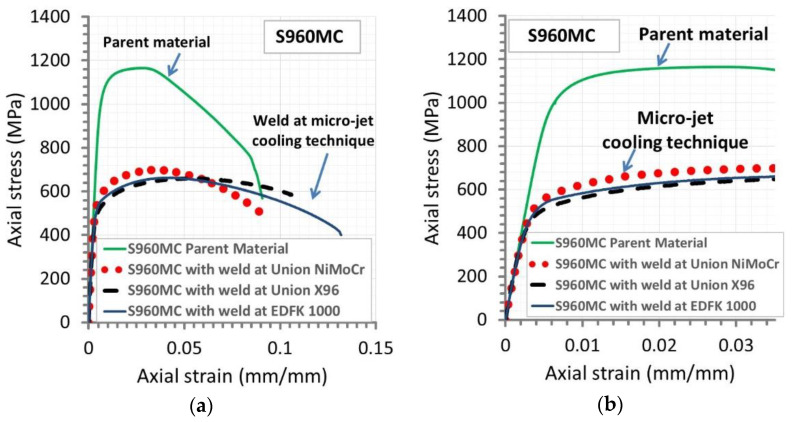
The tensile characteristic of S960MC steel and its weld manufactured using three welding wires at supporting the micro-jet cooling technique: (**a**) up to fracture, (**b**) in a view on the elastic and elastic-plastic relationships.

The steel behavior as a parent material was also dominant compared to welded joints manufactured by the micro-jet cooling technique (Figure 10). The most differences were visible for all stress parameters, placing the steel as a very attractive material compared to its weld. Nevertheless, the data collected for the micro-jet cooled weld enable to indicate advantages of the joint at the loading type considered. As a first, an attractive value of ultimate tensile strength of 650 MPa at all welding wires used can be evidenced. A second feature of the weld is related to a value of relative elongation because, at the fracture, the parameter reached 12.5% for the EDFK wire. This result and a range of the unstable regions enable to formulate sentences related to the application in a tri-axial stress state, i.e., the micro-jet cooled weld behavior after the necking effect is predictable and will be the same for all joints considered. No influence of the welding wire type on the joint’s response was also visible in the elastic and elastic-plastic regions, following almost the same values of stress–strain.

The results from tensile tests collected using mini-specimens have enabled us to follow the stress–strain relationship and mechanical parameters of the S960MC steel. This kind of specimen was also very sufficient at determining the influence of a control signal as displacement, stress, and strain. Literature [17,19,20] indicates mechanical properties of weld for S960MC without any details on a specimen shape and dimensions [17,20] or presents a bigger specimen, i.e., having 1000 mm length [19]. As it was obtained, the steel tested was not sensitive to the signal type used. The mini-specimen has supported examining the small weld, represented by the width of 0.5 mm under static loading, giving a tensile curve. Comparing data from the experiment on the base metal and welds has allowed us to distinguish differences in responses of the regions tested on static loading. Nevertheless, it is worth noting that the applications of small specimens require more attention during the tuning process because the measurement zone with a mounted extensometer is very close to the grips, which at inducing a testing machine can cause total damage to a sensor.

### 3.5. Fatigue Response of the Steel and the Micro-Jet Cooling Weld under Increasing and Constant Stress Amplitude

**Figure 11:**
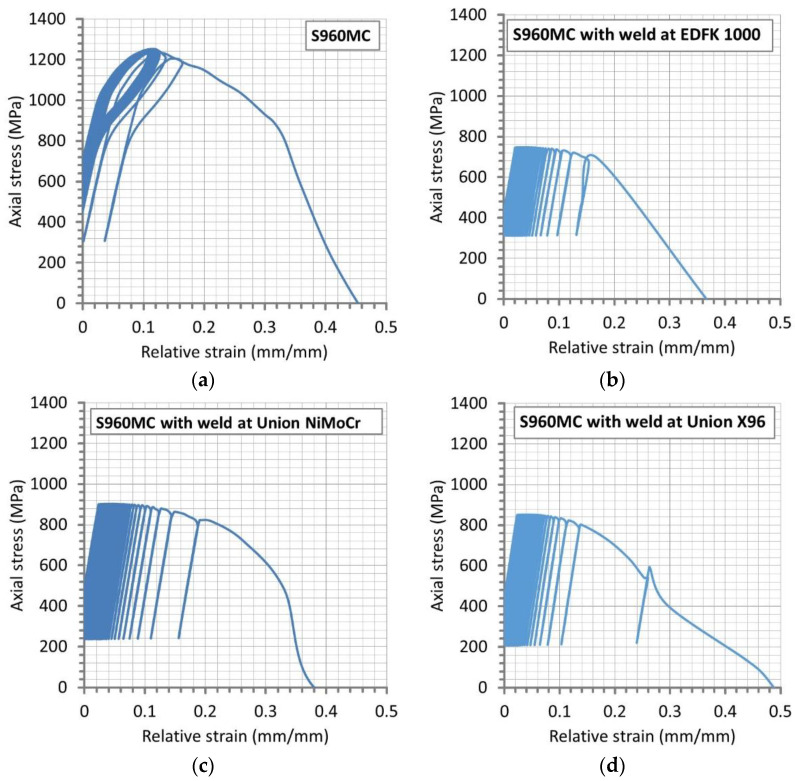
The stress–strain relationship at the final stages of the S960MC steel (**a**) and the joint welded by the micro-jet cooling and the following types of welding wires: (**b**) EDFK 1000, (**c**) Union NiMoCr and (**d**) Union X96.

The behavior of the base material was expressed by hysteresis loops related to the energy dissipation as a dominant response of the material tested on the cycles used (Figure 11a). The first section of the relationship followed the elastic response of the material tested as an effect of hardening due to cyclic loading, while the second one was connected with permanent deformation. As it can be noticed, this feature occurred up to the cycle before the fracture. Moreover, the welds were loaded within the value of stress from the range between yield stress and ultimate tensile strength, and any hysteresis loops were not visible (Figure 11b–d). In this case, the stress–strain relationship has followed ratcheting up to fracture. This was very significant because it increased very rapidly before the fracture. As it can be noticed, this effect was the same for all cases of the welds considered, indicating the behavior of welds manufactured at the micro-jet cooling technique is insensitive to the type of welding wires used.

**Figure 12:**
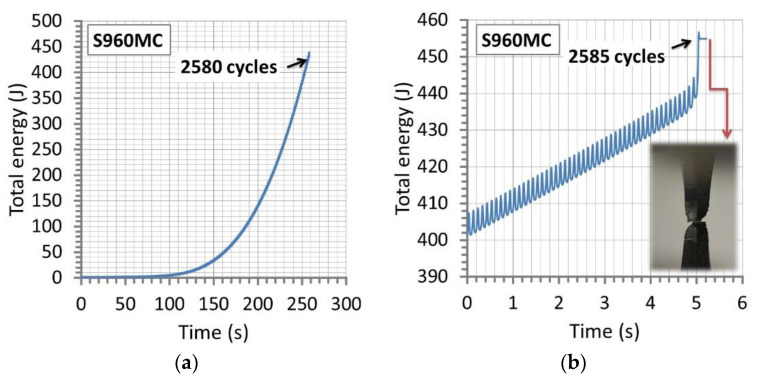
The total energy versus time from tests under increasing amplitude of axial stress before (**a**) and at the fracture (**b**) of S960MC steel, respectively.

**Figure 13:**
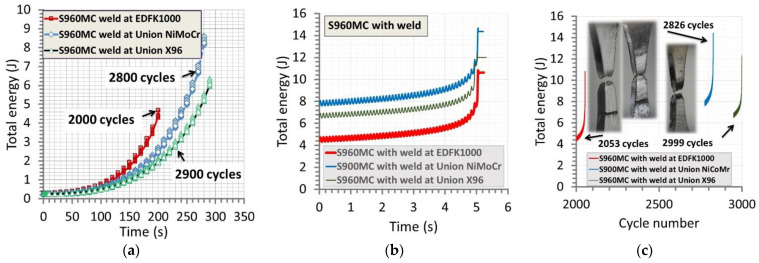
The total energy of the welds versus time (**a**), (**b**) before and at the fracture, respectively, and (**c**) the total energy versus cycle number at fracture for the three welding wires: EDFK 1000, Union NiMoCr and Union X96 for S960MC steel.

The fatigue response of the base material under the increasing value of the stress amplitude was analyzed concerning the courses of total energy (Figure 12 and Figure 13) because it demonstrates data representing loading signal (stress, force) and response signal (strain, elongation, displacement). The values of the physical magnitude were followed from the tests’ beginning up to fracture, focusing on the last 50-th cycles directly related to the degradation of the parent material and the welds. This quantity was calculated basing on features of the Wave Matrix Instron software. It follows the total work performed on the specimen tested [33]. The values of force and displacement were used for calculations of the energy value. This parameter was followed since the start of the test. The software calculates total energy by continuously integrating force with displacement, using the trapezoidal rule [33].

The results collected in the test considered have expressed the same tendency of the values of total energy independently on the region tested, represented by an exponential relationship (Figure 12a and Figure 13a). Differences were visible in courses of the physical quantity, expressing not the same mechanical resistance of the welds on the type of loading applied, Figure 13a. They indicated the weld manufactured at micro-jet cooling technique and EDFK 1000 welding wires had obtained the smallest energy values while the biggest one was represented by results of the micro-jet weld at the NiMoCr wire. The same arrangement of the results was observed on the data directly related to the fracture stages of the regions tested (Figure 13b,c), values of the number of cycles, as well as the time to fracture. In the case of the base material, the value of cycles before the fracture reached 2580 at 258 s (Figure 12b), while for the welds, the following values were obtained: 2000 at 200 s (EDFK 1000 welding wire), 2800 at 280 s (Union NiMoCr welding wire) and 2900 at 290 s (Union X96 welding wire) (Figure 13a).

**Figure 14:**
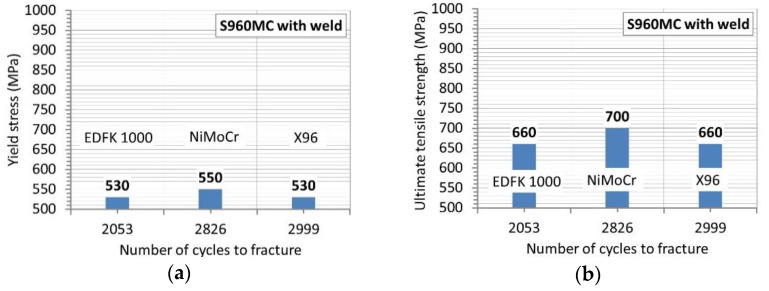

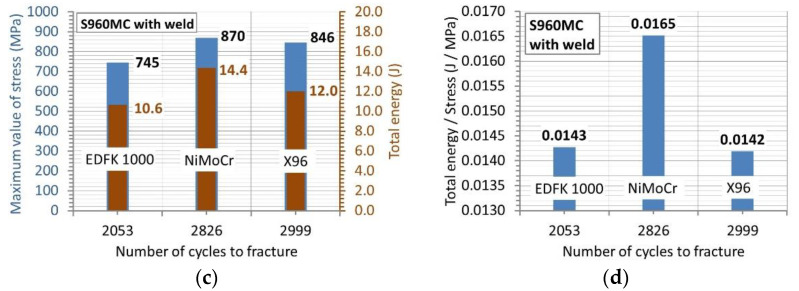
The yield stress (**a**), ultimate tensile strength (**b**), maximum value of stress (**c**) and total energy/the maximum value of stress at a number of cycles (**d**) up to the fracture for welds manufactured by the micro-jet cooling technique and the following welding wires: NiMoCr (Union), EDFK 1000 and X96 (Union).

The results from the fracture stage in the form of the relationship of total energy versus the number of cycles have finally expressed the mechanical resistance of the welds examined (Figure 13c). The smallest one was noticed in the case of the micro-jet cooled weld with EDFK 1000 wire, and the biggest one was observed at the weld produced by the same technique but with the Union X96 wire, reaching the following values, respectively: 2053 and 2999 cycles (Table 7). This result strongly corresponds with the data presented in Figure 13a, which arranges the courses of the total energy in the same order. Some details on the behavior of the region tested can be captured based on the fracture zones. As it can be noticed in the photos presented in Figure 13c, the brittle cracking was the dominant feature of the weld at EDFK 1000; the brittle plastic was related to the weld at Union X96; the plastic represented the behavior of the NiMoCr joint. Nevertheless, any relationships were not observed at the comparison of the values of yield stress, ultimate tensile strength and the number of cycles connected with the fracturing of the tested joints (Figure 14a,b). Some information can be collected based on values of total energy and stress at fracture (Figure 14c,d). This is presented by the proportion of the physical quantity, which arrangements the welds with the EDFK 1000 and Union X96 welding wires as the joints with the same and the lowest mechanical resistance of the loading applied, while the weld of the Union NiMoCr wire become the strongest one from the joint examined.

The results from the tests under increasing stress amplitude were used for selecting the weld for determining the Wöhler curve. For this case, the joint with the Union X96 welding wire as the weakest region welded was tested under cyclic stress at the range of 650 MPa–100 MPa. Data collected from the fatigue experiment are presented in the next sub-chapter.

Fatigue tests at increasing stress amplitude defined by the yield stress and ultimate tensile strength as well as a time have enabled us to distinguish differences in behavior of the welds having very similar tensile characteristics. For this case, the values of the total energy were also significant because they divided into two sections from the beginning of the test up to cycles before and directly at the fracture, giving more information on the regions’ decohesion. This kind of test has allowed us to reach differences in fracture regions, giving more details to assess the mechanical resistance of the weld tested. Values of total energy have supported the analysis on the weld quality expressing the joints response on the cyclic loading. Their comparison with the maximum value of stress of the control signal has enabled to indicate the joints for both welding wires (EDFK 1000 and Union X96) expressed the same proportion, even though the stress levels applied as well as the number of cycles to fracture were significantly different by of 99 MPa and 946 cycles, respectively. Concerning the assessment of the weld quality, the following sentence can be formulated: the joints were manufactured at different welding wires, but nevertheless, they have a very similar reaction to the fatigue process. Taking these results into account, the strongest and weakest joints were possible to be indicated, arranging as of NiMoCr weld, EDFK 1000 and X96 welds.

### 3.6. Results from Tests of the Micro-Cooled Weld under Constant Stress Amplitude

**Figure 15:**
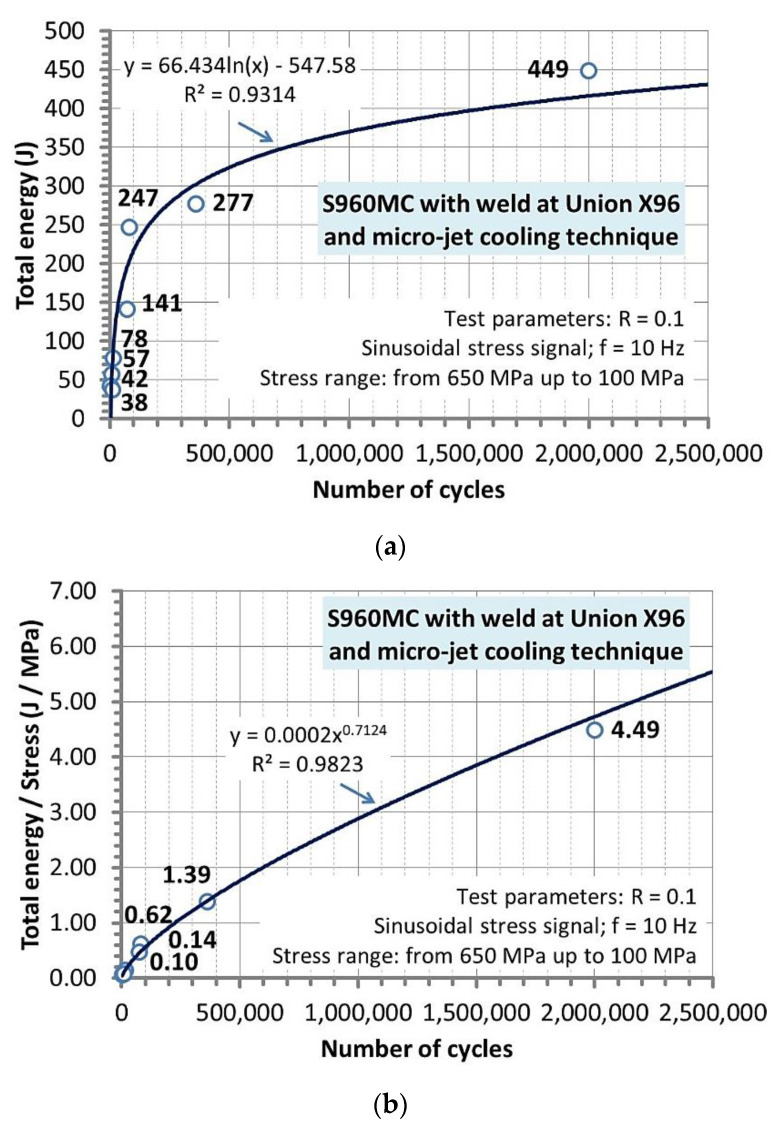
Total energy (**a**) and its value per stress (**b**) versus the number of cycles at the Wöhler curve of S960MC steel welded by the MAG method with the micro-jet cooling technique.

**Figure 16:**
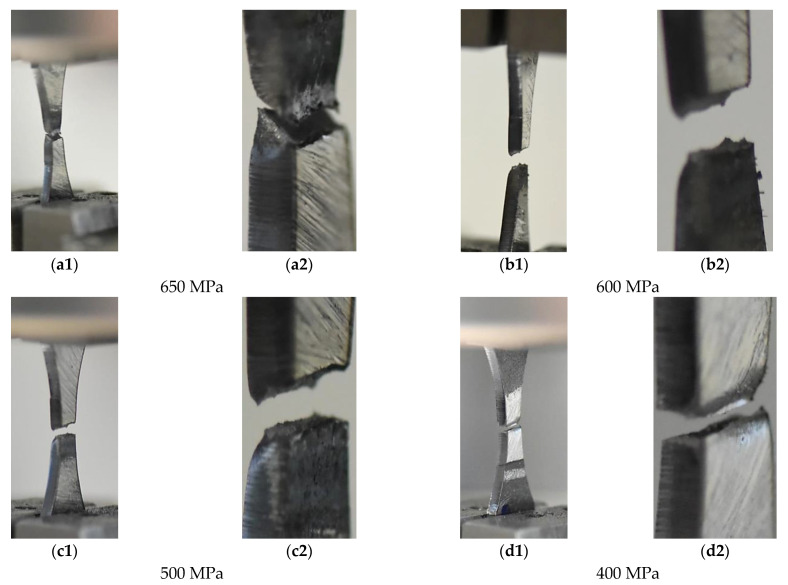

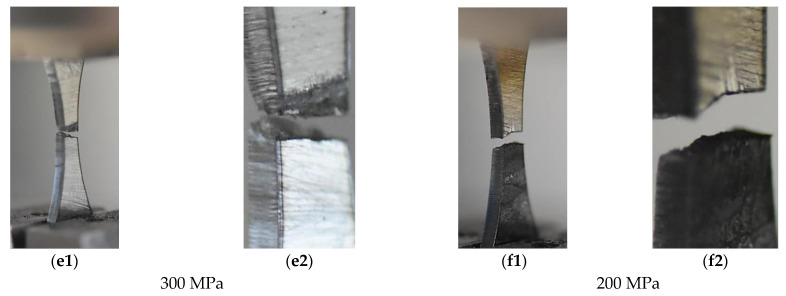
S960MC mini-specimens with the micro-jet cooling weld manufactured with the Union X96 welding wire after the fatigue test for the maximum values of the axial stress amplitude of 650 MPa–100 MPa, (**a1**–**f2**) fracture regions.

**Figure 17:**
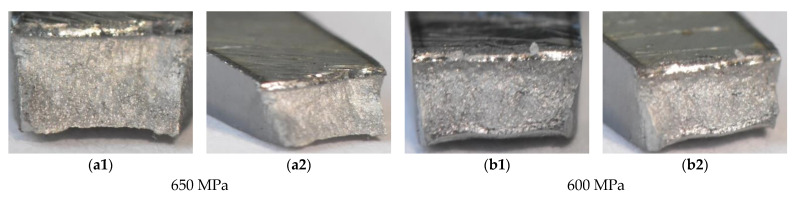

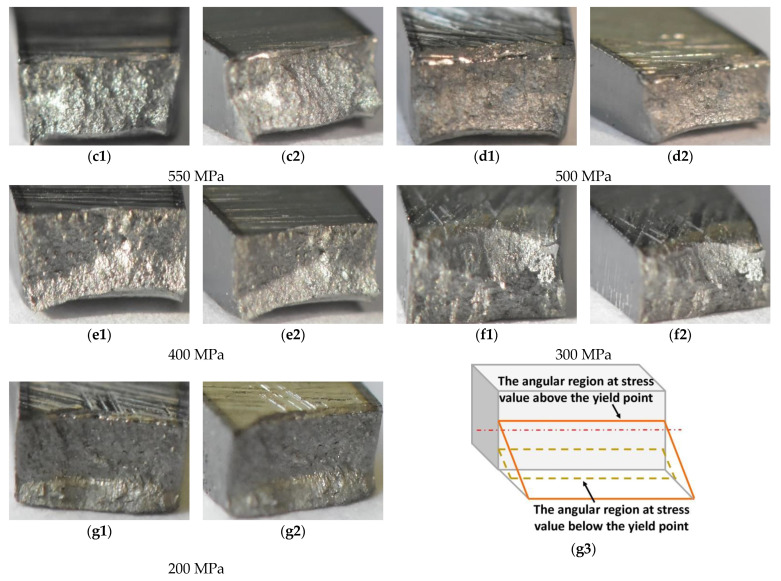
Fracture regions (**a1**–**g3**) of S960MC weld manufactured at micro-jet cooling technique after fatigue tests for the maximum values of axial stress amplitude of 650 MPa–100 MPa and the fracture scheme (**g3**), number 1 represents a perpendicular view while number 2 follows an axonometric one.

**Figure 18:**
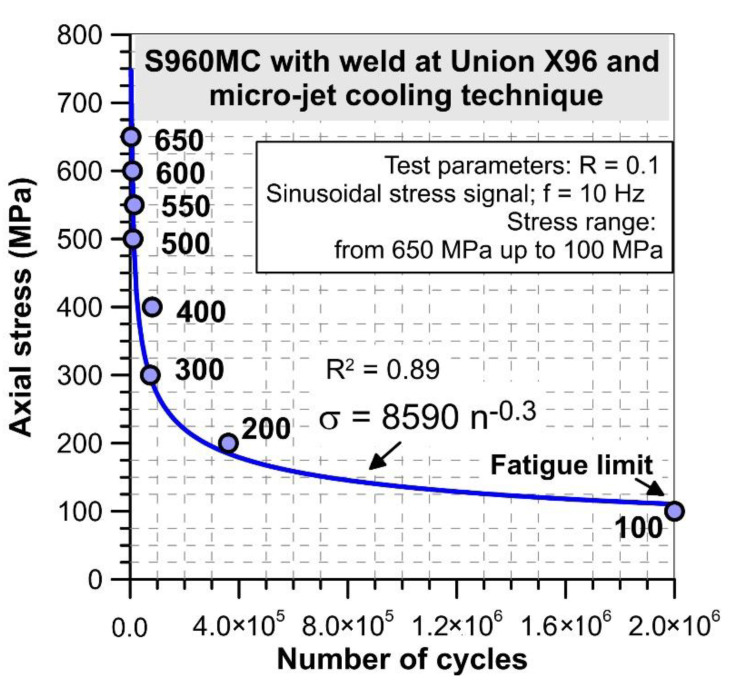
The Wöhler curve of S960MC steel welded by MAG method with the micro-jet cooling technique.

The results from the experiments under stress cycles are represented by variations of total energy (Figure 15a), the proportion of total energy to stress (Figure 15b), fracture regions as both parts of specimen directly after cracking (Figure 16) and as the zones on a perpendicular cross-section of the specimens (Figure 17) as well as the Wöhler curve (Figure 18).

They show a course of total energy up to fatigue limit at values of the number of cycles (Figure 15a). As it can be noticed at the earlier stage of the course up to 5 × 10^5^, the maximum value of total energy is represented by 325 J. For the further section reaching 1 × 10^6^ cycles, the value of 375 J limits, while at the fatigue limit (determined by 2 × 10^6^), an increase of the total energy was expressed by 95 J giving 420 J. If values of total energy and stress are divided then the quantity of Jouls per MegaPascal can be calculated (Figure 15b). This proportion enables to follow the relationship between the physical quantities. In this case, a non-linear tendency was evidenced, indicating the stress values do not have the same influence on the weld fracturing. Moreover, the values of energy can be easily connected with the values of stress (Table 8), covering more practical information for inspection groups. They can be used in comparison with the values of stress as well as total energy from the experiment and captured at operational conditions for predicting the lifetime of components made of the steel grade weld with similar mechanical parameters. The total energy to stress can be used at the current inspection without details on the earlier stage.

The role of the stress level in the weld degradation was determined on fracture zones represented by the general view on the specimens after the test (Figure 16) and exhibited by the view focused on the whole region of degradation (Figure 17). The photos were selected for presenting changes in the fracture regions and for better analysis of the zone degradation at various values of stress. Using them, we can follow the material degradation in a 3D coordinate system and compare it with the orientation of the measured zone. Some differences in the weld degradation due to cyclic loading are visible on the views on both parts of specimens directly after tests (Figure 16). They are expressed by the angular orientation of fracture zones at the stress level of 400–600 MPa, which indicates shear and axial components of stress for the weld decohesion. Comparing these data, a reduction of the angular orientation of the damage region is noticed, indicating the role of shear stress in the fracturing lowers with decreasing the stress value. This is better visible from the cross-section view (Figure 17).

The effect of the booth types of stress in the tested region degradation was also visible on the photos taken with the macro-photography technique (Figure 17). In this case, the same presentation concept for the following decohesion characteristics as was taken for the immediately fracture specimen was used. Using this approach, zone details in relation to fracture mechanics may be easier to collect. The multi-planar cracking was noticed on the perpendicular cross-sections as a major feature of all regions inspected independently of the stress level applied. Nevertheless, the proportion between the angular and horizontal sections of the damage zones was dependent on the values of stress, i.e., the horizontal part becomes more significant with decreasing the stress value (Figure 17g3).

The analysis of the fracture region represented by both zones, i.e., horizontal and angular, enables the selection of the area to be the first for damage occurrence. In this case, the horizontal section of the region considered plays the role of the initial damage. The second area that is damaged further and directly related to the final stage of specimen lifetime is represented by the angular region. This kind of data follows the micro-cooled welded region expresses a mixed (brittle-plastic) cracking at cyclic tensile stress arraigning the axial stress at the beginning of the fracture and shear stress at the final stage of fatigue. In comparison to the good plastic properties of the micro-cooled joint, these results reflect as follows: the weld degradation at plastic cracking is dominant under a stress value exceeding yield stress (530 MPa), while at a smaller one, the brittle behavior appeared.

An analysis of the Wöhler curve has enabled us to select stages with the number of cycles for applications or inspections of the weld tested at a wide range of stress values (Figure 18). For this case, the diagram can be divided at the following regions determined by the stress value and loading cycles: from 650 MPa to 400 MPa at 3 × 10^4^–9 × 10^4^ cycles; from 400 MPa to 100 MPa at 9 × 10^4^–2 × 10^6^ cycles. This enables to formulate as follows: the weld expresses hardening due to cyclic tensile stress close to the ultimate tensile strength reaching the lifetime limited by the 3 × 10^4^. Moreover, the value of fatigue limit can be indicated as 100 MPa, which unfortunately does not express an attractive level for application under cyclic loading at very restricted operational regimes. This kind of result was very similar to data collected by the authors of [19]. Nevertheless, their data did not follow the function proposed as it was obtained in the paper. A comparison of these test results shows that they are complementary due to the different characteristics of the zones containing the welds and can be used for extending the knowledge in the S960MC weld behavior.

Concluding results from tests on the Wöhler curve worth noticing are that this kind of data can be analyzed not only at variations stress versus a number of cycles but also at changes of total energy. This approach collects the relationship between control and response signal, i.e., stress and strain, respectively, supporting results represented by one of the mentioned physical quantities. This kind of data can be used for modifying models for predicting lifetimes as well as elaborating on new ones. Fracture analysis is the stage of the fatigue test for analysis of fracture mechanics due to the type of loading applied. This follows the orientation of a fracture plane compared to a loading direction as well as details related to brittle or brittle-plastic cracking. The first mentioned feature of the region subjected to observation should be captured directly after a specimen fracturing without unmounting the tested object from the grips of the testing machine, while the second one’s details are obtained from observations of a fracture plane from various directions. As it was evidenced in the photos of the fracture regions of the weld, the degradation mechanism was related to stress state components, i.e., the shear stress becomes more significant in the weld degradation with an increasing stress level becoming from a value close to the yielding point.

## 4. Summary

The article deals with MAG welding of thin-walled structures made of S960MC steel. It is a material used in automotive and offshore engineering [34]. Various welding parameters were tested, including micro-jet cooling parameters. To assess the quality of the joints, a series of non-destructive and destructive tests were performed. Welds were made with three different electrode wires and two different shielding gas mixtures. Main welding parameters and parameters of micro-jet cooling were varied. After the results of non-destructive tests, the main information was obtained on the proper welding parameters, where no cracks appeared. The results of non-destructive tests showed that the use of micro-jet cooling is very beneficial, as there were no welding cracks. The condition for a properly made joint was the correct selection of micro-jet cooling parameters. Then bending tests were performed. The results of these tests showed that in addition to cooling the micro-jet, the selection of the shielding gas is important. The use of the Ar gas mixture is beneficial, while the use of CO_2_ does not guarantee the possibility of obtaining joints with good plastic properties. Further research focused on the evaluation of joints made with three different welding wires. M21 cover and micro-jet cooling were always used. The U-notched specimen supports experiments for assessment of the quality of the welding process. Moreover, the specimen size was small, and all mechanical parameters of the static and fatigue behavior of the material and joints tested were determined. The results from the static and fatigue tests have extended the knowledge on the weld testing, the role of the three types of welding wires for the joint’s quality manufactured by the micro-jet cooling technique, as well as applications and inspections. This was represented by variations of the stress-strain relationship, total energy directly at fracture and versus the number of cycles up to fatigue limit.

## 5. Conclusions

The results from the non-destructive and destructive experiments have enabled the following conclusions:

- The S960MC steel is very difficult for welding processes even when high technology is used, such as the micro-jet cooling method, and when different welding wires are applied because the weld’s mechanical properties can be lowered by 50%;
- The following welding parameters are recommended for MAG-welding sheet metal with a thickness of 2 mm: U = 19 V, I = 101 A, V = 300 mm/min and a mixture Ar-18% CO_2_ as shielding gas;
- Elongation (10.50%) of the weld produced at controlling micro-jet cooling with the three welding wires exceeded the value of this parameter (8.75%) captured at the test of the base metal;
- At the same type of mechanical parameters, such as yield stress and ultimate tensile strength, their values were almost the same for all cases of the welds tested, i.e., 537 MPa and 673 MPa, respectively. An increasing stress amplitude enabled following the differences in the behavior of the joints even they represent very similar response in static tests;
- The weld manufactured with the Union NiMoCr welding wire was the joint with the highest resistance on static and fatigue loadings. The response of the weld under cyclic loading should be analyzed not only in the general form represented by the Wöhler curve but also variations of total energy;
- As it was determined in the test, the value of energy at the fatigue limit reached 449 J. In the case of the micro-jet cooled weld with the Union X96 welding wire, the difference between the value of stress from limited and unlimited sections was six times, following the value of fatigue limit of 100 MPa at the final stage of the last-mentioned section;
- The use of micro-jet cooling when welding S960MC steel allows for obtaining joints with better mechanical properties (UTS = 700 MPa, YS = 550 MPa).

## Figures and Tables

**Table 1:** The welding current of S960MC steel.

Diameter of the Electrode, mm	Current Intensity, A	Voltage, V	Polarization + or −	Welding Speed, mm/min
1.0	101	19	DC “+”	300

**Table 2:** The characteristic of the carried tests.

No	Test	Characteristic of Test
**I**	**Non-Destructive Tests:**	
1.	Visual testing (VT)	was performed with an eye armed with a loupe (Levenhook, Tampa, FL, USA) at 3× magnification and the test was made using standard auxiliary measures, luxmeter with white light 520 Lx;
		✓ tests were carried out in accordance with the requirements of the PN-EN ISO 17638 standard,
		✓ evaluation criteria according to the EN ISO 5817 standard.
2.	Magnetic-particle test	was made using the wet method with the following conditions: field strength 3 kA/m, white light 515 Lx, temperature 20 °C, MR-76 detection means, MR-72 contrast; the device for testing was a magnetic flaw detector of REM-230 type (ATG, Prague, Czech Republic);
		✓ the tests were carried out in accordance with the PN-EN ISO 17638 standard,
		✓ the evaluation of the tests was carried out in accordance with the EN ISO 5817 standard.
**II**	**Destructive Tests:**	
3.	The bending test (BT)	was carried out using the ZD-40 testing machine (WPM, Leipzig, Germany);
		✓ tests was carried out in accordance with the PN-EN ISO 5173 standard, using the ZD-40 testing machine (WPM, Leipzig, Germany), (The tests used: specimens with a thickness of a = 2 mm, width b = 20 mm, mandrel d = 22 mm, spacing of supports d + 3a = 31 mm, and the required angle of bending 180. Five bending test measurements were carried out for each tested joint thickness on the root side and on the face side).
4.	Examination of microstructure	was made using the of specimens etched with Adler reagent using light microscopy (Neophot 32, Carl Zeiss Jena, Jena, Germany);
		✓ the tests were carried out in accordance with the PN-EN ISO 9016 2021 standard.
5.	Microhardness—Vickers method	was carried out using the Zwick/Roell ZHV-30S hardness tester (ZwickRoell GmbH, Ulm, Germany);
		✓ the tests were carried out in accordance with the PN-EN ISO 6507-01:2018-05, the hardness was measured 3 times in all tested zones of a joint.
6.	Tensile and fatigue tests	8874 Instron servo-hydraulic testing machine (Instron, High Wycombe, UK) and mini-specimens at room temperature;
		✓ the tests were carried out in accordance with the PN-EN ISO 6892-1:2020 and ASTM E468-18, respectively.

**Table 3:** Details in the form of mechanical parameters of the welds to experiment under increasing stress amplitude.

No.	Specimen	Yield Stress (MPa)	Ultimate Tensile Strength (MPa)	Time for the Envelop (s)
1.	Weld at EDFK 1000	530	660	600
2.	Weld at Union NiMoCr	550	700	
3.	Weld at Union X96	530	660	

**Table 4:** The assessment of non-destructive testing on the movable platform joint.

Designation	Shielding Gas	Wire, C Amount (%)	Micro-Jet Stream Pressure (MPa)	Micro-Jet Stream Diameter (µm)	Micro-Jet Gas	Observation Acceptability: (**×** or **✓**, B) *
UNm1	Ar + 18% CO_2_	Union NiMoCr, (0.08)	without	without	without	×
UNm2	Ar + 18% CO_2_	Union NiMoCr, (0.08)	0.6	60	Ar	×
UNm3	Ar + 18% CO_2_	Union NiMoCr, (0.08)	0.7	60	Ar	✓, B
UNm4	Ar + 18% CO_2_	Union NiMoCr, (0.08)	0.6	70	Ar	✓, B
UNm5	Ar + 18% CO_2_	Union NiMoCr, (0.08)	0.7	70	Ar	×
EDm1	Ar + 18% CO_2_	ED-FK 1000, (0.09)	without	without	without	×
EDm2	Ar + 18% CO_2_	ED-FK 1000, (0.09)	0.6	60	Ar	×
EDm3	Ar + 18% CO_2_	ED-FK 1000, (0.09)	0.7	60	Ar	✓, B
EDm4	Ar + 18% CO_2_	ED-FK 1000, (0.09)	0.6	70	Ar	✓, B
EDm5	Ar + 18% CO_2_	ED-FK 1000, (0.09)	0.7	70	Ar	×
UXm1	Ar + 18% CO_2_	Union X96, (0.11)	without	without	without	×
UXm2	Ar + 18% CO_2_	Union X96, (0.11)	0.6	60	Ar	×
UXm3	Ar + 18% CO_2_	Union X96, (0.11)	0.7	60	Ar	✓, B
UXm4	Ar + 18% CO_2_	Union X96, (0.11)	0.6	70	Ar	✓, B
UXm5	Ar + 18% CO_2_	Union X96, (0.11)	0.7	70	Ar	×
UNc1	CO_2_	Union NiMoCr, (0.08)	without	without	without	×
UNc2	CO_2_	Union NiMoCr, (0.08)	0.6	60	Ar	×
UNc3	CO_2_	Union NiMoCr, (0.08)	0.7	60	Ar	×
UNc4	CO_2_	Union NiMoCr, (0.08)	0.6	70	Ar	✓, B
UNc5	CO_2_	Union NiMoCr, (0.08)	0.7	70	Ar	×
EDc1	CO_2_	ED-FK 1000, (0.09)	without	without	without	×
EDc2	CO_2_	ED-FK 1000, (0.09)	0.6	60	Ar	×
EDc3	CO_2_	ED-FK 1000, (0.09)	0.7	60	Ar	✓, B
EDc4	CO_2_	ED-FK 1000, (0.09)	0.6	70	Ar	×
EDc5	CO_2_	ED-FK 1000, (0.09)	0.7	70	Ar	×
UXc1	CO_2_	Union X96, (0.11)	without	without	without	×
UXc2	CO_2_	Union X96, (0.11)	0.6	60	Ar	×
UXc3	CO_2_	Union X96, (0.11)	0.7	60	Ar	×
UXc4	CO_2_	Union X96, (0.11)	0.6	70	Ar	×
UXc5	CO_2_	Union X96, (0.11)	0.7	70	Ar	×

* Board description: ✓, B—No cracks in the weld, level of quality-B without welding defects and incompatibilities; ×—Cracks in the weld.

**Table 5:** Bending tests results.

Sample	Deformed Side	Observation *
UNm3	root	✓
UNm3	face	✓
UNm4	root	✓
UNm4	face	✓
EDm3	root	✓
EDm3	face	✓
EDm4	root	✓
EDm4	face	✓
UXm3	root	✓
UXm3	face	✓
UXm4	root	✓
UXm4	face	✓
UNc4	root	×
UNc4	face	×
EDc3	root	×
EDc3	face	×

* Board description; ✓: No cracks in the weld, without welding defects and incompatibilities; ×: Cracks in the weld.

**Table 6:** The hardness of the joint (UXm3).

MAG Process	Base Material			HAZ			Weld		
With m-j cooling	334	336	337	354	349	351	327	329	332
Without m-j cooling	338	335	339	371	369	373	341	342	344

**Table 7:** The results from tests under increasing stress amplitude.

No.	Specimen	Axial Stress at Fracture (MPa)		Time to Fracture (s)	Number of Cycle to Fracture
		Maximum	Minimum		
1.	Parent material	1253	306	269	2585
2.	Weld at EDFK 1000	745	314	216	2053
3.	Weld at Union NiMoCr	870	229	293	2826
4.	Weld at Union X96	846	214	310	2999

**Table 8:** Values of stress, total energy and their proportion.

No.	Maximum Value of Stress (MPa)	Total Energy (J)	Total Energy/Maximum Value of Stress (J/MPa)
1.	650	42	0.06
2.	600	57	0.10
3.	550	78	0.14
4.	500	38	0.08
5.	400	247	0.62
6.	300	141	0.47
7.	200	277	1.39
8.	100	449	4.49

## Data Availability

Data sharing is not applicable to this article.
